# Syntabulin regulates neuronal excitation/inhibition balance and epileptic seizures by transporting syntaxin 1B

**DOI:** 10.1038/s41420-023-01461-7

**Published:** 2023-06-22

**Authors:** Pingyang Ke, Juan Gu, Jing Liu, Yan Liu, Xin Tian, Yuanlin Ma, Yuan Meng, Fei Xiao

**Affiliations:** 1 Department of Neurology, The First Affiliated Hospital of Chongqing Medical University, Chongqing Key Laboratory of Neurology, Chongqing, 400016 China; 2 Department of Neurology, The Affiliated Traditional Chinese Medicine Hospital of Southwest Medical University, Luzhou, 646000 China

**Keywords:** Epilepsy, Genetics of the nervous system

## Abstract

Epilepsy is a widespread neurological disorder affecting more than 65 million people, but the mechanisms of epilepsy remains unknown. Abnormal synaptic transmission has a crucial role in the occurrence and development of epilepsy. Here, we found that syntabulin, a neuronal transporter, was mainly localized in neurons, and its expression was increased in epileptic tissues. Knockdown of syntabulin increased susceptibility and severity of epilepsy, whereas overexpression of syntabulin had the opposite effect. Mechanistically, in the epileptic brain tissue, syntabulin mainly translocated syntaxin 1B (STX1B) rather than syntaxin 1A (STX1A) to the presynaptic membrane, which resulted in increased presynaptic transmitter release. Further studies showed that syntabulin had a more significant effect on presynaptic functionality of GABAergic activity over that of excitatory synapses and resulted in an excitation/inhibition (E/I) imbalance, thereby regulating the epileptic phenotype. In addition, we found that the increased expression of syntabulin in epileptic brain tissue was mainly regulated by transcription factor TFAP2A. In summary, syntabulin plays a protective role in epilepsy by maintaining a proper E/I balance in the hippocampus.

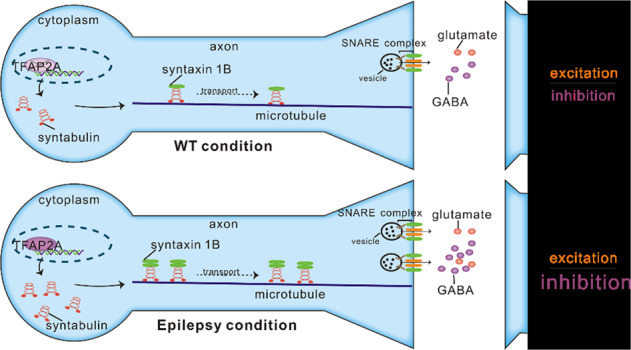

## Introduction

Epilepsy is a neurological disease characterized by recurrent seizures. More than 65 million people worldwide are affected by epilepsy [1, 2]. 70 percent of these people can control their seizures with antiepileptic drugs, but about 30 percent of these people cannot and instead develop drug-resistant epilepsy [3, 4]. Drug-resistant epilepsy results in psychological and cognitive disorders to patients, leading to heavy economic and social burden [5]. The most common form of drug-resistant epilepsy is temporal lobe epilepsy (TLE), therefore there is an urgent need to explore new mechanisms of TLE pathogenesis and drug targets.

The pathogenesis of epilepsy is complex. Currently, it is widely accepted that epilepsy is related to excitation/inhibition (E/I) imbalance in the central nervous system [6]. There are many factors leading to E/I imbalance, among which the most important factor is synaptic transmission. Dysregulation of presynaptic neurotransmitter release and postsynaptic receptors may lead to E/I imbalance, which may underline or exacerbate epilepsy progression [6, 7]. The expression of presynaptic proteins on the surface and at synaptic sites are often closely related to the release of presynaptic neurotransmitters and are key determinants of synaptic strength [8]. The newly synthesized presynaptic proteins in the cell body of the neuron must be transported along the axon to the presynaptic terminal to function [9]. Dysregulation of the mechanisms that mediate the transport of these presynaptic proteins occurs in many neurological disorders, including epilepsy [10].

Syntabulin, as a molecular motor adapter, is a microtubule-associated protein [11, 12]. Syntabulin is expressed in the nervous system, including the mouse hippocampal cell layer [13]. Syntabulin binds to kinesin family member 5B (KIF 5B) and mediates the transport of active zone (AZ) components along microtubules to the presynaptic membrane to determine presynaptic assembly [14]. Thus, syntabulin maintains presynaptic function and mediates synaptic transmission [12]. Syntaxin 1 (STX1) localizes to the plasma membrane of neurons and forms soluble N-ethylmaleimide-sensitive factor attachment protein receptor (SNARE) complexes with SNAP-25 and synaptobrevin to mediate vesicle docking and neurotransmitter release [10, 15]. STX1 has two forms: STX1A and STX1B. STX1A and STX1B are highly homologous, and their cDNA are 84% identical, illustrating that they have similar functions [15, 16]. As previously described, syntabulin acts as a linker molecule to connect STX1A-containing vesicles to microtubules, allowing STX1A to be transported along the axon to presynaptic terminals [11].

In proteomic analysis of postmortem cortices from patients with Alzheimer’s disease, dementia with Lewy bodies and Parkinson’s disease, syntabulin expression changes significantly [17]. Furthermore, syntabulin knockout mice develop an autistic phenotype due to impaired presynaptic cargo transport, reduced synaptic density and active zones, and altered synaptic transmission [18]. These results demonstrate that syntabulin is strongly associated with neurological diseases; however, whether syntabulin affects epilepsy and its underlying mechanisms remains unclear.

Here, using biochemical, behavioral, and electrophysiological approaches, we show that syntabulin plays a protective role in epilepsy by interacting with STX1B, but not STX1A, and mediating its delivery to the synapse. Furthermore, syntabulin-dependent STX1B trafficking and synaptic delivery is critical for presynaptic neurotransmitter release and E/I balance, which protects the brain from epilepsy and may be a potential therapeutic target.

## Results

### Expression and localization of syntabulin in epileptic tissues

**Fig. 1:**
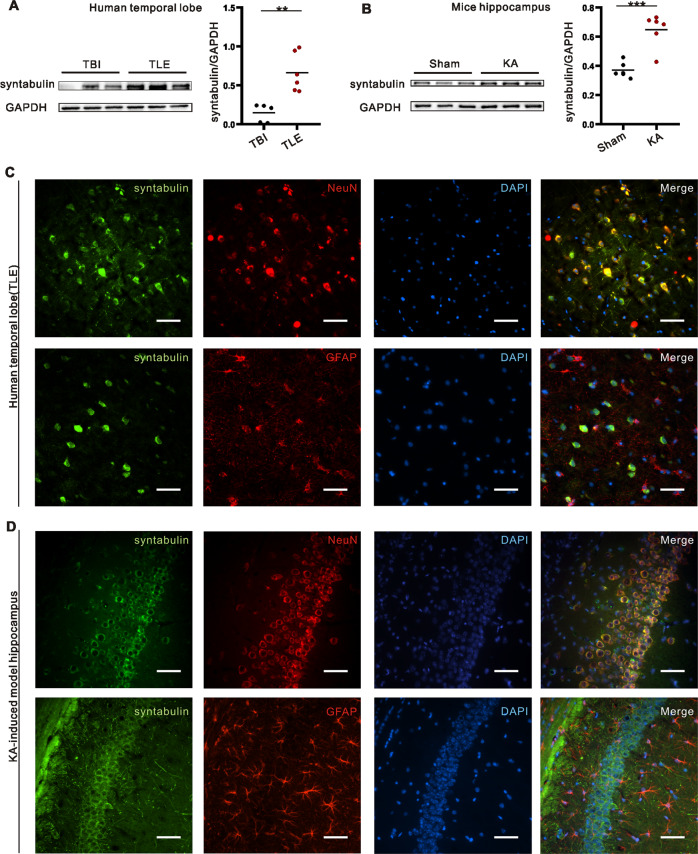
Expression and localization of syntabulin in epileptic tissues. **A** Increased expression of syntabulin protein in the cerebral cortex of TLE patients (*n* = 5 in TBI group, *n* = 6 in TLE group). **B** Syntabulin protein levels in the hippocampus of mice with KA-induced epilepsy. **C**, **D** Syntabulin colocalizes with NeuN but not GFAP in cortex of TLE patients and hippocampus of KA epileptic mice. Scale bars: 50 μm. **p < 0.01, ***p < 0.001, unpaired two-tailed Student’s *t*-test (**A**, **B**).

To explore the role of syntabulin in epilepsy, we first examined the expression of syntabulin in TLE patients and kainic acid (KA) mouse model. Western blotting (WB) showed that syntabulin was significantly upregulated in TLE patients compared with non-epileptic traumatic brain injury (TBI) patients (Fig. 1A, Supplementary Fig. 2A). Similarly, the same results were observed in the KA mouse model (Fig. 1B, Supplementary Fig. 2B). In addition, syntabulin colocalized with neuronal nuclear protein (NeuN, neuronal marker) but not glial fibrillary acidic protein (GFAP, astrocyte marker) (Fig. 1C, D) in the cortex of TLE patients and hippocampal CA1 region of epileptic mice. These results suggest that the expression and localization of syntabulin in epileptic patients and mice may be related to epilepsy.

### Syntabulin alterations affect seizure susceptibility and severity

**Fig. 2:**
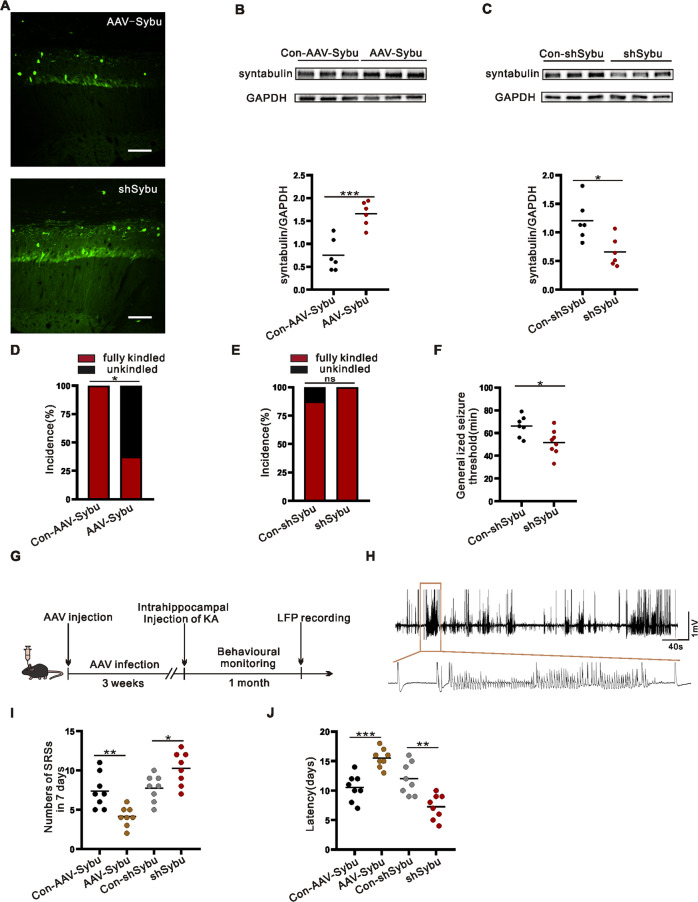
Syntabulin affects seizure susceptibility and severity. **A** Fluorescence signal of hippocampal CA1 region infected with AAV-Sybu or shSybu. Scale bars: 100 μm. **B**, **C** Syntabulin protein expression infected with AAVs (*n* = 6/group). **D**, **E** Kindled rates in AAV-Sybu and shSybu mice injected with PTZ (*n* = 8/group). **F** Seizure threshold in shSybu mice injected with PTZ. (*n* = 8/group). **G** Experimental design of KA-induced TLE model. **H** Typical LFP recording during spontaneous seizures in KA-induced epileptic mice. **I**, **J** SRSs numbers in the last 7 days and SRSs latency (*n* = 8/group). ns means *p* > 0.05, *p < 0.05, **p < 0.01, ***p < 0.001, Pearson Chi-square test (**D**, **E**), unpaired two-tailed Student’s *t*-test (**B**, **C**, **F**, **I**, **J**).

To investigate whether syntabulin influences epilepsy, AAV-Sybu (syntabulin overexpression) and shSybu (syntabulin knockdown) were injected into CA1 region of mice, and pentylenetetrazol (PTZ) or KA modeling and behavioral testing were performed 3 weeks later. We confirmed effective infection via immunofluorescence stainings of the hippocampal region 3 weeks after AAV injection (Fig. 2A). Meanwhile, WB indicated that, compared with the corresponding control group, the AAV-Sybu group and the shSybu group effectively increased and inhibited the expression of syntabulin (Fig. 2B, C, Supplementary Fig. 2C and 1D). Therefore, syntabulin expression was distinctly altered by the corresponding AAV injection.

Next, we tested the effect of syntabulin on susceptibility to epilepsy in mice. In the PTZ model, the incidence of fully kindled mice in the AAV-Sybu group was lower than that of the Con-AAV-Sybu group (Fig. 2D), while knockdown of syntabulin had the opposite effect (Fig. 2E). Furthermore, the latency was shorter in the shSybu group than in the Con-shSybu group (Fig. 2F). Altogether, changing the expression of syntabulin had a significant effect on the susceptibility of PTZ mice.

Spontaneous recurrent seizures (SRSs) are an important feature of epilepsy, and we next examined whether altering syntabulin affects SRSs. There are showed on the KA mouse model-related behavioral monitoring time axis (Fig. 2G) and local field potential (LFP) signal in the hippocampal region (Fig. 2H). During the last 7 days of behavioral monitoring, overexpression of syntabulin decreased the number of SRSs compared with the Con-AAV-Sybu group. However, compared with the Con-shSybu group, the number of SRSs in the shSybu group was significantly increased (Fig. 2I). Overexpression of syntabulin increased SRSs latency, but knockdown of syntabulin had the opposite effect (Fig. 2J). At the same time, western blot results showed that AAVs still significantly affected syntabulin expression after video monitoring. AAVs interventions maintained the entire behavioral cycle (Supplementary Fig. 1A, B).These findings revealed that syntabulin expression affects SRSs in KA mouse model.

### Syntabulin binds and transports more STX1B than STX1A

**Fig. 3:**
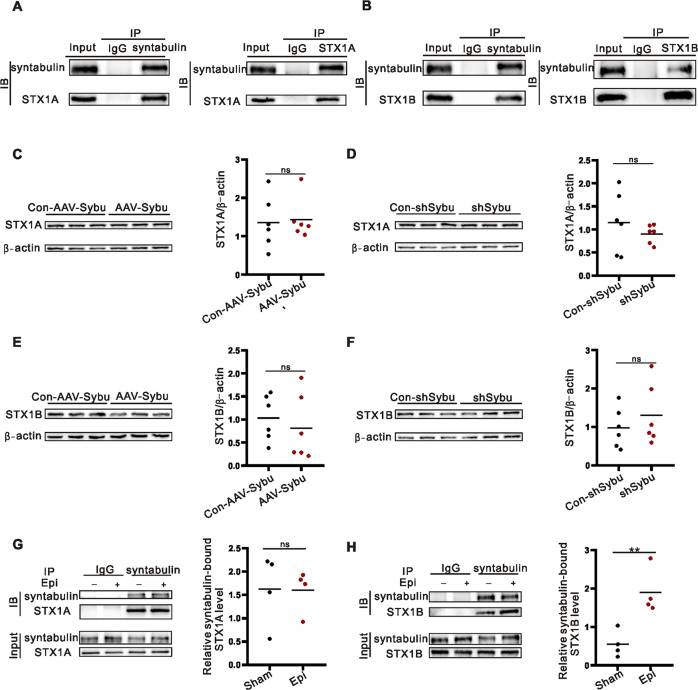
Syntabulin binds more STX1B in epilepsy. **A**, **B** The binding of syntabulin to STX1A/1B by CO-IP assay. **C**–**F** AAVs don’t affect the total expression of STX1A or STX1B protein (*n* = 6/group). **G**, **H** The amount of syntabulin bound to STX1A/1B by quantitative CO-IP (*n* = 4/group). ns means *p* > 0.05, **p < 0.01, unpaired two-tailed Student’s *t*-test (**C**–**H**).

We next queried the potential mechanisms by which syntabulin affects epilepsy. In the hippocampus of KA model mice, syntabulin bound STX1A or STX1B according to Co-Immunoprecipitation(CO-IP) assay (Fig. 3A, B, Supplementary Fig. 3A, B). At the same time, overexpression or knockdown syntabulin did not cause changes in the total expression of STX1A and STX1B (Fig. 3C–F, Supplementary Fig. 3C, D, Supplementary Fig. 4A, B). Subsequently, in the quantitative CO-IP assay, we found that syntabulin bound more STX1B in the epilepsy group than in the corresponding control group (Fig. 3H, Supplementary Fig. 4D), but the amount of syntabulin bound STX1A did not differ (Fig. 3G, Supplementary Fig. 4C). These experiments suggested that syntabulin may affect epilepsy by transporting more STX1B rather than affecting STX1B expression.

### Syntabulin regulates STX1B expression at synaptosome

**Fig. 4:**
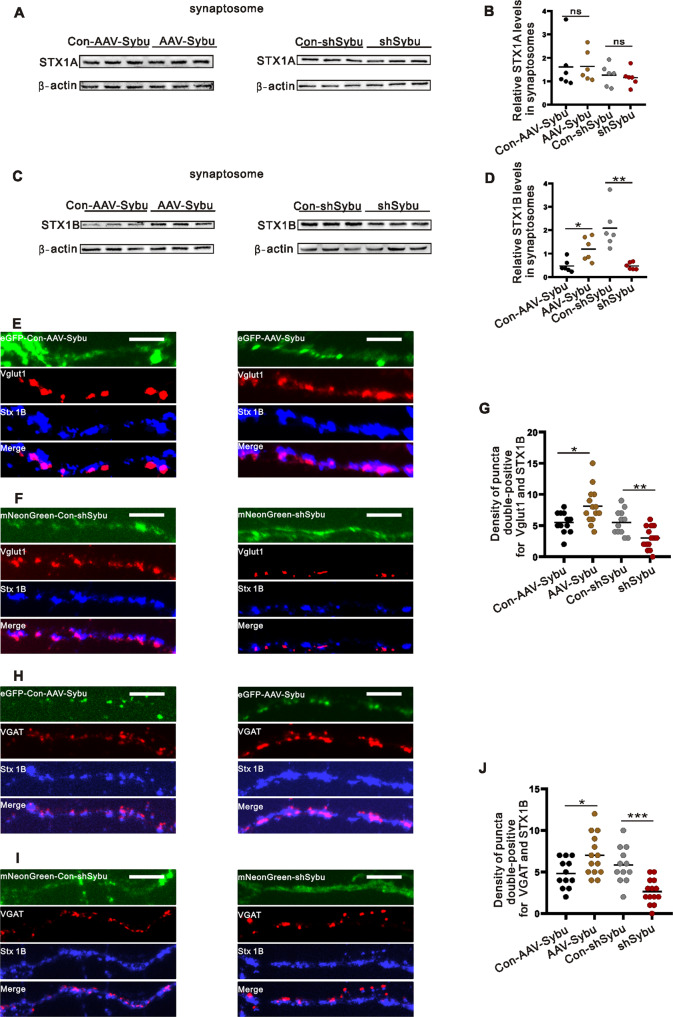
Syntabulin transports more STX1B to the synaptosome. **A**–**D** Representative immunoblotting images of STX1A/1B expression in synaptosomes (*n* = 6/group). **E**–**J** Colocalization of STX1B and Vglut1/VGAT fluorescence signal and statistical analysis (Con-AAV-Sybu group=12 cells, AAV-Sybu group = 14 cells, Con-shSybu group = 12 cells, shSybu group = 14 cells). Scale bars = 10 μm. ns means *p* > 0.05, *p < 0.05, **p < 0.01, ***p < 0.001, unpaired two-tailed Student’s *t*-test (**B**, **D**, **G**, **J**).

Since STX1 forms SNARE complexes in synaptosomes to mediate neurotransmitter release, to calculate STX1A/1B expression in synaptosomes regulated by syntabulin, hippocampal synaptosomes were extracted from KA model mice after AAV intervention to detect the expression of STX1A/STX1B. The results displayed that STX1B expression was increased in synaptosomes in the AAV-Sybu group compared with the Con-AAV-Sybu group. Conversely, knockdown of syntabulin decreased STX1B expression in synaptosomes (Fig. 4C, D, Supplementary Fig. 4F), but STX1A is not affected (Fig. 4A, B, Supplementary Fig. 4E). To further investigate syntabulin modulate STX1B expression at excitatory and inhibitory synapses, we performed immunofluorescence assay on primary neurons after AAV intervention and found that STX1B expression was increased at excitatory or inhibitory synapses in the AAV-Sybu group compared with that in the Con-AAV-Sybu group (Fig. 4E, G, H, J). However, knockdown of syntabulin reduced STX1B expression at excitatory or inhibitory synapses (Fig. 4F, G, I, J). Thus, these results indicated that syntabulin regulates STX1B expression at excitatory and inhibitory synapses.

### Syntabulin affects presynaptic function

**Fig. 5:**
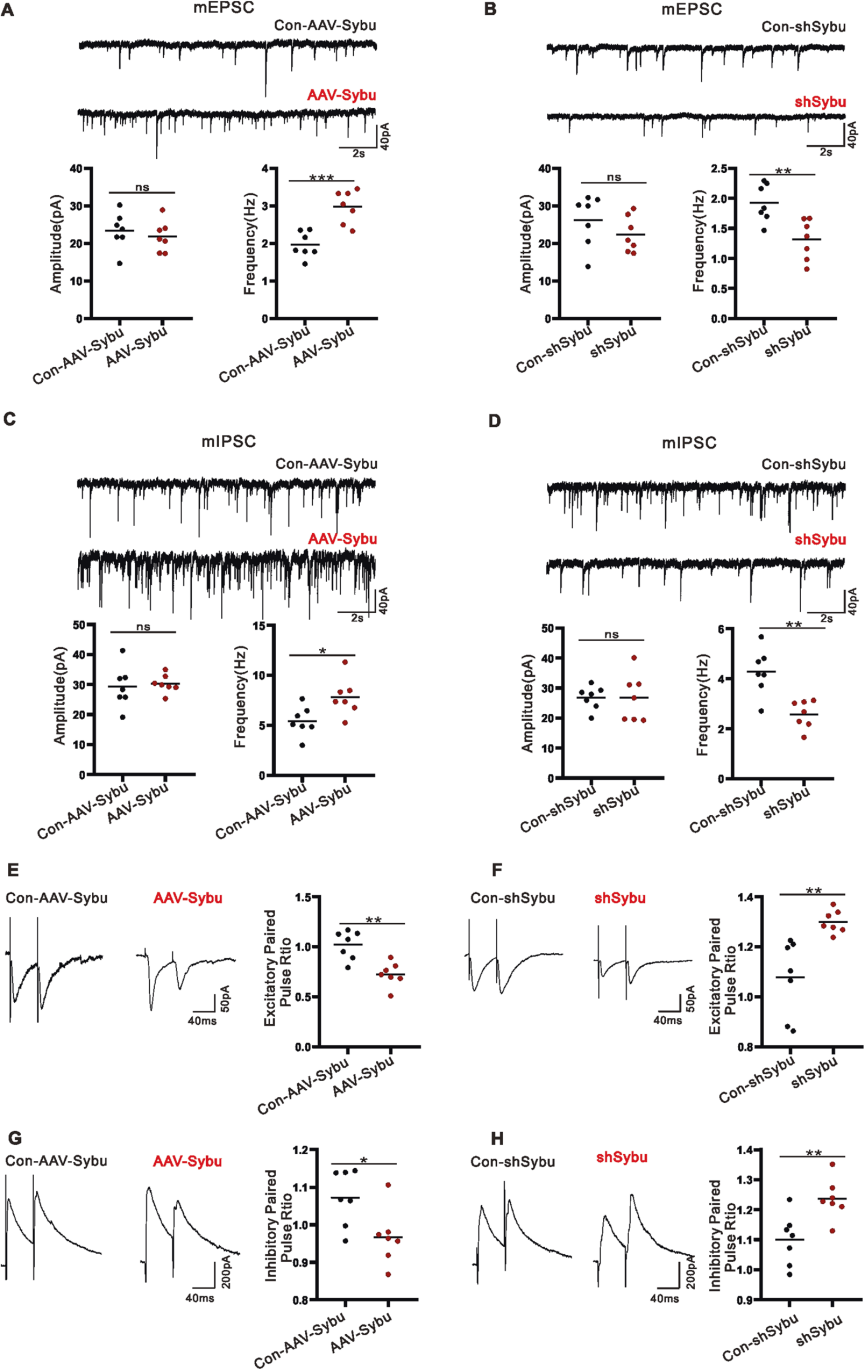
Syntabulin affects presynaptic function. **A**, **B** Representative traces and statistical analysis of mEPSC, *n* = (7 cells, 4 mice)/group. **C**, **D** Representative traces and statistical analysis of mIPSC, *n* = (7 cells, 4 mice)/group. **E**–**H** The PPR for representative traces and statistical analysis, *n* = (7 cells, 4 mice)/group. ns means *p* > 0.05, *p < 0.05, **p < 0.01, ***p < 0.001, unpaired two-tailed Student’s *t*-test (**A**–**H**).

To investigate whether syntabulin modulates synaptic transmission, we tested the miniature excitatory postsynaptic currents (mEPSCs) and miniature inhibitory postsynaptic currents (mIPSCs) in the CA1 region of hippocampal slices of KA model mice after syntabulin AAVs intervention. Compared with Con-AAV-Sybu group, the frequency of mEPSC and mIPSC in the AAV-Sybu group increased, but the amplitude was not different (Fig. 5A, C). Meanwhile, compared with the Con-shSybu group, the frequency of mEPSC and mIPSC in the shSybu group decreased, but the amplitude was not different (Fig. 5B, D). Therefore, the frequency changes of mEPSCs and mIPSCs induced by syntabulin are consistent with the changes caused by STX1B [19, 20], which both lead to presynaptic changes, including excitatory and inhibitory neurons. To further understand the effect of syntabulin on synaptic transmission, we further detected the paired pulse ratio (PPR) for EPSCs and IPSCs in hippocampus brain slices of KA model mice after AAVs intervention. Compared with Con-AAV-Sybu group, PPR for EPSCs and IPSCs were reduced in AAV-Sybu group (Fig. 5E, F). However, knockdown of syntabulin increased the PPR compared with Con-shSybu group (Fig. 5G, H). These results had shown that syntabulin modulate presynaptic function in excitatory/inhibitory neurons.

### Syntabulin regulates neural excitability

**Fig. 6:**
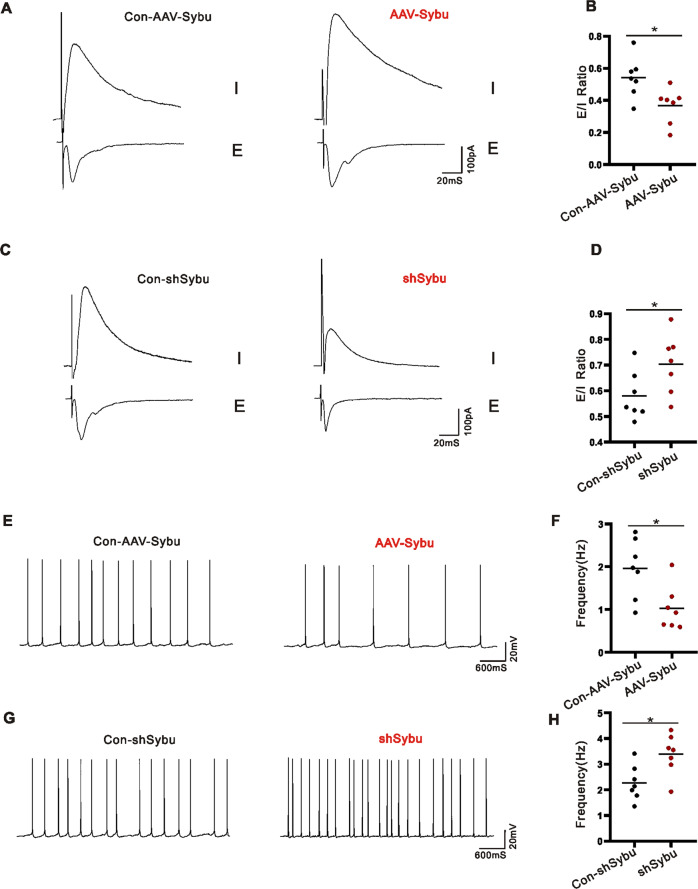
Syntabulin regulates neural excitability. **A**–**D** Representative E/I traces and quantitative analysis of the E/I ration, *n* = (7 cells, 4 mice)/group. **E**–**H** Representative traces and statistical analyses of sAPs, *n* = (7 cells, 4 mice)/group. *p < 0.05, **p < 0.01, unpaired two-tailed Student’s *t*-test (**B**, **D**, **F**, **H**).

Epilepsy is a kind of disease characterized by imbalance of E/I. Because syntabulin affect the synaptic transmission of excitatory and inhibitory neurons, we evaluated the E/I ratio in KA model mice after syntabulin AAVs intervention. We observed that the E/I ratio was decreased in the AAV-Sybu group compared with the Con-AAV-Sybu group (Fig. 6A, B). However, compared with Con-shSybu group, the E/I ratio of shSybu group was increased (Fig. 6C, D). To further assess neural excitability changes, spontaneous action potentials (sAPs) were recorded. Overexpression of syntabulin reduced the frequency of sAPs (Fig. 6E, F), and knockdown of syntabulin had the opposite effect (Fig. 6G, H). These findings indicated that syntabulin significantly changes neural excitability and is associated with epilepsy.

### TFAP2A regulates syntabulin expression

**Fig. 7:**
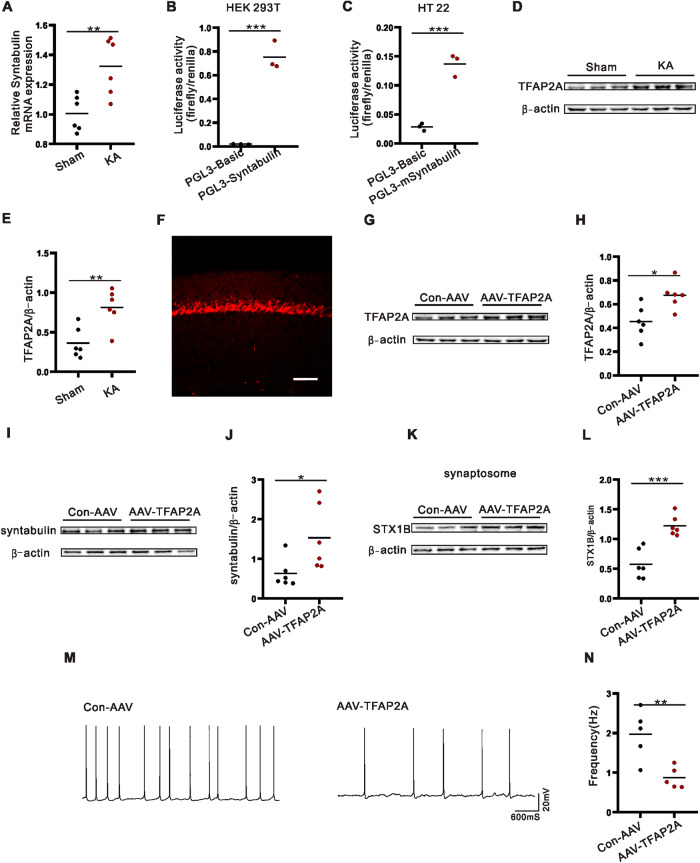
TFAP2A regulates syntabulin expression. **A** Syntabulin mRNA expression detection by PCR (*n* = 6/group). **B**, **C** Dual luciferin reporter assay is used to detect syntabulin transcriptional regulation by TFAP2A (*n* = 3/group). **D**, **E** TFAP2A protein expression of WB images and statistical analysis (*n* = 6/group). **F**–**H** Fluorescence signal in hippocampus infected with AAV-TFAP2A (**F**). and AAV-TFAP2A significantly increases TFAP2A protein expression (**G**, **H**). **I**–**L** AAV-TFAP2A increases overall syntabulin expression (*n* = 6/group) (**I**, **J**) and STX1B expression in synaptosomes (*n* = 6/group) (**K**, **L**). **M**, **N** Overexpression of TFAP2A decreases neural excitability (*n* = 5/group). *p < 0.05, **p < 0.01, ***p < 0.001, unpaired two-tailed Student’s *t*-test (**A**–**C**, **E**, **H**, **J**, **L** and **N**).

To further determine the upstream mechanism of syntabulin, we tested the increase of *syntabulin* mRNA expression in the hippocampus of KA model mice by qPCR (Fig. 7A). Next, the upstream transcription factor- transcription factor activating enhancer binding protein 2 α (TFAP2A), which is highly matched with syntabulin and notably related to epilepsy [21], was predicted by JASPAR software. Dual luciferase reporter assay was applied to verify that TFAP2A acted on the promoter of *syntabulin* gene and regulated *syntabulin* gene transcription (Fig. 7B, C). To examine the role of TFAP2A in epilepsy, we detected increased TFAP2A expression in the hippocampus of KA model mice (Fig. 7D, E, Supplementary Fig. 5A). This is a fluorescent signal of AAV-TFAP2A infection in the hippocampus (Fig. 7F). Additionally AAV-TFAP2A significantly increased TFAP2A expression (Fig. 7G, H, Supplementary Fig. 5B). In the hippocampus of KA model mice after AAV-TFAP2A intervention, syntabulin expression was increased (Fig. 7I, J, Supplementary Fig. 5C) and STX1B expression was increased in synaptosomes (Fig. 7K, L, Supplementary Fig. 5D) compared with Con-AAV-TFAP2A group. At the same time, the frequency of sAPs in the AAV-TFAP2A group was lower than that in the Con-AAV-TFAP2A group (Fig. 7M, N). These evidences suggest that TFAP2A regulates syntabulin expression in epilepsy and STX1B expression in synaptosomes, and affects neural excitability.

## Discussion

The communication between neurons depends on the fusion and release of neurotransmitters in the presynaptic terminals, and these processes are subtly regulated. The abnormal regulation will lead to the dysregulation of neurotransmitter release, which is involved in the pathogenesis of nervous system diseases including epilepsy [6, 7]. The SNARE complex composed of synaptobrevin, STX1 and SNAP-25 is the core presynaptic mechanism that mediates vesicle fusion [10]. A large number of studies have reported that abnormalities in SNARE proteins are closely related to epilepsy [22], and that these presynaptic proteins, along with transport from cell body synthesis to presynaptic transmission, are necessary to maintain normal synaptic transmission [9]. Our current study identified a protective role for the microtubule-associated protein syntabulin in epilepsy and revealed for the first time the molecular mechanism of syntabulin-dependent STX1B synaptic trafficking in maintaining E/I balance in epilepsy.

A recent study found that mice with syntabulin specifically knocked out of the central nervous system exhibited core symptoms similar to autism [18]. These results suggest that the expression of syntabulin may be related to some central nervous system diseases. This hypothesis is confirmed by our study, which provides evidence that syntabulin protein expression is elevated in brain tissue from epilepsy patients and animal models of epilepsy and plays a compensatory protective role. Knockdown of syntabulin resulted in the aggravation of the epileptic phenotype, which further suggested that the altered expression of syntabulin plays an important role in epilepsy. The increase of syntabulin protein level may be regulated at the transcriptional level or post-transcriptional level. We found that syntabulin increased in the brain tissue of epilepsy animal model, suggesting that syntabulin may be regulated by upstream transcription factors. According to JASPAR software prediction and verification, we found that syntabulin is regulated by transcription factor TFAP2A in epilepsy. Previous studies also found that TFAP2A expression is significantly increased in epileptic hippocampal RNA sequencing analysis [21]. Consistent with these findings, we detected increased TFAP2A expression in epileptic brain tissue. Whether other transcription factors are also involved in the regulation of syntabulin transcription needs further study. In addition, whether other potential mechanisms such as epigenetic modifications also affect syntabulin mRNA expression in epilepsy cannot be ruled out. In conclusion, our results suggest that syntabulin is transcriptionally activated in epilepsy and subsequently involved in the development of epilepsy.

Previous studies have shown that syntabulin interacts with STX1A and that syntabulin acts as a linker molecule, binding KIF5B to transport STX1A along microtubules [11]. Therefore, we asked whether syntabulin affects epilepsy by regulating the synaptic trafficking of STX1A/1B, since STX1B and STX1A are highly homologous and both are major syntaxin family members mediating the release of presynaptic transmitters [15, 16], and the functional abnormalities of both are related to epilepsy [23, 24]. Therefore, we examined the interaction of syntabulin with STX1A and STX1B in epileptic brain tissue. Interestingly, we found that syntabulin interacts not only with STX1A but also with STX1B in vivo. Further study showed that syntabulin did not affect the total protein expression of the two proteins. Unexpectedly, syntabulin significantly increased STX1B, but not STX1A expression in synaptosomes in epileptic brain tissue. To our knowledge, the synaptic transport of STX1B by syntabulin was first discovered in this study. Numerous studies have shown that missense mutations in STX1B cause a wide spectrum of epileptic disorders [19, 24–30]. STX1B missense mutations are distributed throughout the protein but concentrated in conserved SNARE motifs [24]. This may be due to mutations in STX1B affecting SNARE complex formation and abnormal presynaptic transmitter release followed by seizures. In line with to these findings, our results suggest that syntabulin positively regulates the localization and expression of STX1B at synapses and subsequently affects synaptic transmission to participate in the development of epilepsy. However, why syntabulin only affects the synaptosomal transport of STX1B in epileptic brain tissues deserves further study.

Abnormal release of presynaptic neurotransmitters in the central nervous system is closely related to epilepsy [6]. In the mammalian brain, glutamate is the major excitatory neurotransmitter, while GABA is the major inhibitory transmitter. The release of these neurotransmitters is finely regulated to maintain normal synaptic transmission [31]. SNARE complexes can mediate membrane fusion in different cell types. Different SNARE protein compositions play different functions in different cells [32]. Previous data have established a model of how presynaptic proteins mediate the release of neurotransmitters. In this model, a tight complex formed by STX1, SNAP-25, and synaptobrevin is essential for neurotransmitter release [33]. As an important isoform of STX1, we found that syntabulin positively regulates STX1B expression at both excitatory and inhibitory synapses, which is consistent with previous reports that STX1B is expressed at both excitatory and inhibitory synapses [34]. The increased binding of syntabulin to STX1B in the epileptic hippocampus and the increased expression of syntabulin in the synaptosome suggested that there may be more SNARE complex formation in the presynaptic membrane, which leads to the increased release of excitatory and inhibitory presynaptic transmitters.

Altered presynaptic transmitter release may cause excitation/inhibition imbalance leading to epilepsy [6, 35]. We found that syntabulin positively regulates the frequency of mEPSC and mIPSC but does not change their amplitude. This suggests that syntabulin may regulate excitatory and inhibitory presynaptic neurotransmitter release. In addition, syntabulin negatively regulated EPSC-PPR and IPSC-PPR, further indicating that syntabulin positively regulates excitatory and inhibitory presynaptic neurotransmitter release evoked by action potentials. The regulation of presynaptic transmitter release by syntabulin may be mediated by STX1B, which is consistent with previous reports of an increase in PPR caused by generative STX1B knockdown [20]. It is well accepted that the E/I imbalance causes epilepsy. Our study shows that the high expression of syntabulin in epileptic brain tissue leads to increased inhibitory drive compared to excitatory drive, which inhibits synaptic transmission in the hippocampus. This suggested that the increase of syntabulin in epileptic brain tissue is essential to maintain proper synaptic transmission by adjusting the release of neurotransmitters.

Overall, we show for the first time that syntabulin transits more STX1B (but not STX1A) to the excitatory/inhibitory presynaptic membrane in epilepsy, suppressing neuronal hyperexcitability. TFAP2-syntabulin-STX1B axis is a critical pathway in epilepsy, thus may provide a potential new target for epilepsy treatment and drug development.

## Materials and methods

### Human brain tissue and animal samples

All the brain tissues of patients with TLE (Supplementary Table 1 and Table 2) or TBI were obtained from the First Affiliated Hospital of Chongqing Medical University during surgery. Patients with TLE were definitely diagnosed according to the International League Against Epilepsy [36]. TBI patients had no history of neuropsychiatric problems. Patients and their families signed informed consent forms and participated in scientific studies. All male C57BL/6 J mice (age 8 weeks, weight 20–25 g) were obtained from the Laboratory Animal Center of Chongqing Medical University. All mice were kept in Specific Pathogen Free (SPF) standard environment (temperature: 20 ~ 26 °C, light/dark time: 12/12 h, adequate feed and water). The use of all samples was approved by the Ethics Committee of Chongqing Medical University and the First Affiliated Hospital of Chongqing Medical University. The number of all samples was determined by sample calculation. And the assignment of mice to each group was random. The specific number of samples for each group is shown in “Figure Legends”.

### Construction of adeno-associated virus and intrahippocampal injection

A short hairpin RNA with the target sequence 5 ‘-GCCTACACAGAAGAAAGAT-3’ was carried by the adeno-associated virus vector (pAAV-U6-shRNA/spgRNA v2.0-CMV-EGFP-WPRE). It was designated to shSybu, which decreases syntabulin expression in the hippocampus. Adeno-associated virus vector (pAAV-CMV-3xFLAG-P2A-mNeonGreen-tWPA) containing full-length syntabulin cDNA was used to express syntabulin and was designated as AAV-Sybu. The control viruses Con-AAV-Sybu and Con-shSybu were same with the promoter of AAV-Sybu and shSybu. All AAVs were purchased from Obio Biotechnology Co., Ltd. (Shanghai, China).

1 μl viral particles were injected into the bilateral hippocampus via microsyringe (Hamilton, Reno, USA) (coordinates, anteroposterior: −2.0 mm, mediolateral: ±1.5 mm,dorsoventral: −1.6 mm; Injection speed: 200 nl/min), then the needle was stayed on each side for 10 min after injection to prevent reflux.

### Epilepsy models and LFP recording

C57BL/6 mice(6–8 weeks) were injected with PTZ (10 mg/kg, Sigma-Aldrich, MO, USA) intraperitoneally every 10 min for 8 times after virus particle intervention. Seizure rating was assessed according to the Racine scale [37]. When the seizure grade reached grade IV, the seizure latency and kindled number were recorded.

In the KA model, mice were anesthetized with pentobarbital (1%) and fixed in stereotaxic instrument (RWD Life Science Co.Ltd., Shenzhen, China). 1.0 nmol KA (1.0 nmol in 50 nl saline, Sigma-Aldrich, St. Louis, MO, USA) was injected into the unilateral hippocampus (coordinates, anteroposterior: −2.0 mm, mediolateral: 1.5 mm, dorsoventral: −1.5 mm; injection rate: 100 nl/min). After the KA injection, recovery from anesthesia was awaited. Mice SRSs were then monitored for 30 days. The time of the first spontaneous seizure (grade IV or above, Racine scale) and the number of SRSs in the last 7 days were recorded. Finally, LFP recording was performed according to the previous description [38].

We randomly extracted the hippocampus of mice(after behavioral monitoring) for some molecular biology experiments. Since not all experiments could be satisfied, we created epileptic mice with the same intervention pattern, and then conducted the remaining molecular biology experiments (verification of STX1A/1B expression in synaptosomes) and electrophysiological experiments.

### Western blot

Tissue samples were lysed with RIPA lysate and centrifuged (16000 g, 4 °C) to obtain the supernatant. Synaptosome proteins were obtained according to the instruction (SY-052, Invent Biotechnologies, Minnesota, USA). The protein is separated in SDS-PAGE gel (10%) and then transferred to polyvinylidene fluoride (PVDF) (0.45 um) membrane. After transferring, the membrane was blocked for 10 min by using protein free rapid blocking buffer (Epizyme Biomedical Technology, Shanghai, China), and then incubated with primary antibody at 4 °C overnight. The next day, the membrane was washed 3 times with Tris-buffered saline containing 0.1% Tween-20 (TBST) for 5 min each. Then, the membrane was incubated with secondary antibody (Proteintech, catalog SA00001-2, Wuhan, China) for 1 h and washed for 5 min/3 times. Membranes were imaged using the Fusion Imaging System. The following primary antibodies were used in the western blot: rabbit anti-syntabulin (1: 1000, Proteintech, Cat No: 16972-1-AP), rabbit anti-syntaxin 1 A (1: 1000, Synaptic Systems, Cat. No.: 110 302, Goettingen, Germany), rabbit anti-syntaxin 1B (1: 1000, Synaptic Systems, Cat. No.: 110 402), rabbit anti-TFAP2A (1:500, Abcam, ab108311, Cambridge, UK), rabbit anti-GAPDH (1: 5000; Proteintech; Cat No. 10494-1-AP), rabbit anti-beta actin (1:5000, Proteintech, Cat No: 81115-1-RR).

### Immunofluorescent staining

To prepare frozen sections for immunofluorescence, after the mice were euthanized, they were perfused with PBS and paraformaldehyde (PFA) (4%). Mice brain tissues were immobilized with PFA (4%) overnight and dehydrated with sucrose (30%) for 48 h. The brain tissue was sectioned using a cryotome. Brain sections were permeated with Triton-X100 (0.3%) for 20 min at 37 °C and then fixed with goat serum (10%) for 1 h. Sections were incubated with primary antibody overnight at 4 °C. The following primary antibodies were used in tissue immunofluorescence staining: anti-syntabulin (Proteintech, Cat No: 16972-1-AP), anti-NeuN (Millipore, NBP192693PE, Massachusetts, USA),anti-GFAP (Abcam, ab4648). The sections were washed for 3 times and incubated with secondary antibody in PBS for 1 h[DyLight 488, goat anti-rabbit IgG (Abbkine, A23220, Wuhan, China); DyLight 594, goat anti-mouse IgG (Abbkine, A23410)]. For primary neurons, cells were treated with sucrose (4%) and PFA (4%) dissolved in PBS for 30 min at room temperature. Primary neurons were permeated with Triton-X100 (0.1%) for 10 min at 37 °C and then blocked with goat serum (10%) for 1 h. Neurons were incubated with primary antibodies overnight at 4 °C. The following primary antibodies were used in cell immunofluorescence staining: anti-syntaxin 1B (Synaptic Systems, Cat. No.: 110 402), anti-Vglut1 (Synaptic Systems, Cat. No.: 135 304), anti-VAGT (Synaptic System, Cat. No.: 131 011). Neurons were washed 3 times in PBS and incubated with secondary antibody for 1 h [Dylight 594, Goat Anti-Rabbit IgG (Abbkine, A23420), DylightTM 405-conjugated AffiniPure Goat Anti-Guinea Pig IgG (Jackson ImmunoResearch, 147530, PA, USA), DyLight 405, Goat Anti-Rabbit IgG (Abbkine, A23120), DyLight 594, goat anti-mouse IgG (Abbkine,A23410)]. Images were captured by using immunofluorescence microscopy. ImageJ was used for colocalization analysis.

### Immunoprecipitation

The mice hippocampi were cleaved and used for immunoprecipitation. According to the instructions for protein A/G magnetic beads (MCE, Cat. No. HY-K0202, Washington, USA), mice hippocampal lysates were incubated at 4 °C for 2 h with antibodies (anti-syntabulin, anti-syntaxin 1 A, anti-syntaxin 1B). The proteins on the isolated beads were analyzed by Western blot analysis after washing.

### Primary neuron culture

Brain tissue from mice (0 day) was removed and then separated into cell suspensions by trypsin (0.25%) for 30 min at 37 °C. The solution was transferred to DMEM medium containing 10% FBS. Primary neurons were plated on glass slides pretreated with Poly-L-Lysine (0.5 mg/kg) and then added to neurobasal medium (2% B27, 0.05 M L-glutamine). These primary neurons were maintained at 37 °C in a cell incubator containing 5% CO_2_. Half of the medium was replaced every 3 days.

### Section preparation and whole-cell patch-clamp recording

After the mice were euthanized, the brain tissue was isolated to prepare brain slices. The brain tissue was cut into 300 µm sections using frozen sectioning fluid[95% O_2_, 5% CO_2_, 1.25 mM NaH_2_PO_4_; (in mM): 2.5 KCl, 2 CaCl_2_, 2 MgCl_2_, 26 NaHCO_3_, 220 sucrose, 10 D-glucose]. Then the brain tissue sections were recovered at 32 °C for 30 min with artificial cerebrospinal fluid (ACSF) [PH = 7.4; (in mM) 2.5 KCl, 119 NaCl, 26 NaHCO_3_, 1.25 NaH_2_PO_4_, 2 CaCl_2_, 1 MgCl_2_, 25 D-glucose].

For recording mEPSCs, a glass electrode was contained with internal fluid[PH = 7.4, 280–290 mOsm; (in mM): 130 CsMeSO_4_, 10 CsCl, 4 NaCl, 10 HEPES, 1 MgCl_2_, 5 MgATP, 1 EGTA, 0.5 Na_3_GTP, 5 NMG, 12 phosphocreatine], and the cell voltage was maintained at −70 mV. Tetrodotoxin (TTX) (1 μM) and picrotoxin (100 μM) were added in external ACSF.

mIPSCs was measured with a glass electrode containing an internal fluid[PH = 7.4; 280–290 mOsm; (in mM): 1 EGTA, 1 MgCl_2_, 100 CsCl, 5 MgATP, 10 HEPES, 0.5 Na_3_GTP, 30 N-methyl-D-glucamine (NMG), 12 phosphocreatine]. TTX (1 μM), D-2-amino-5-phosphonovaleric acid (D-AP5, 50 μM), and 6,7-dinitroquinoxaline-2,3 (1H, 4H) -dione (DNQX, 20 mM) were added, and the cell voltage was maintained at −70 mV.

PPR at excitatory synapses was recorded by holding the patched hippocampal CA1 pyramidal neurons at −70 mV in external ACSF with PTX (100 μM). The internal solution used was the same as that used for mEPSC recording. PPR at inhibitory synapses was measured by holding the patched hippocampal CA1 pyramidal neurons at 0 mV in external ACSF with D-AP5 (50 μM) and DNQX (20 μM). Paired stimuli interval was set at 50 ms and delivered at 0.1 Hz frequency to stimulate the Schaffer collateral axon tracts. PPR was measured as the ratio of the second amplitude peak to the first peak.

E/I ratio recordings were performed in external ACSF with no inhibitors. The internal solution used was the same as that used for mEPSC recording. Evoked EPSCs were recorded at −60 mV, while the evoked IPSCs were recorded at 0 mV. A single pulse of voltage was delivered at 0.4 Hz to stimulate the Schaffer collateral axon tracts.

sAPs were recorded with glass electrodes containing internal fluid[(in mM): 0.5 BAPTA, 2 Na_2_ATP, 12 phosphocreatine, 0.2 Na_3_GTP, 60 N-methyl-D-glucamine4 MgCl_2_·6H_2_O, 40 HEPES, 60 K_2_SO_4_].

### qPCR

RNAsimple Total RNA Kit (Tiangen, Beijing, China) was used to extract total RNA from mouse hippocampus. cDNA was obtained according to the instructions of the kit (Vazyme, R223-01, Nanjing, China). qPCR was applied by using ChamQ SYBR qPCR Master Mix kit (Vazyme, Q341-02). The following primers are used in qPCR:

mouse syntabulin(F): TACGCCCCTTCTTCTCCAAG

mouse syntabulin(R): CCGTGATTTTCGCCACAAGA

mouse GAPDH(F): CAGTGGCAAAGTGGAGATTGTTG

mouse GAPDH(R): TCGCTCCTGGAAGATGGTGAT

### Double luciferase reporter assay

According to the LipofectamineTM 3000 reagent (Invitrogen, CA, USA) operator’s instructions, pGL3-Basic (91.4 ng/μl) or Sybu promoter pGL3-Basic (73.8 ng/μl), TFAP2A pcDNA 3.1 (100.4 ng/μl), pRL-TK (106.7 ng/μl) were co-transfected into HEK 293 T cells (Shanghai Institutes for Biological Sciences of Chinese Academy of Sciences, Shanghai, China). pGL3-Basic (91.4 ng/μl) or mSybu promoter pGL3-Basic (73.7 ng/μl), mTFAP2A pcDNA 3.1 (94.3 ng/μl), pRL-TK (106.7 ng/μl) were co-transfected into HT 22 cells(Shanghai Institutes for Biological Sciences of Chinese Academy of Sciences, Shanghai, China). After 24 h, luciferase activity was measured according to the Dual-Lumi™ Luciferase Reporter Gene Assay Kit (Beyotime, RG088S, Jiangsu, China) instructions.

### Statistical analysis

GraphPad Prism 8 was used for statistical analyses. Results of data are presented via mean ± standard deviation (SD). *p* < 0.05 was considered statistical difference. Enumeration data with multiple categories of two or more factors were analyzed via Pearson Chi-square test. When the two groups of data conformed to the normal distribution, the unpaired *T*-test is carried out. In case of uneven variances, an unpaired *t*-test with Welch’s correction was performed. When the sample value was greater than 3 times the standard deviation, it was regarded as an outlier and would be removed. All tests and statistical analyses were performed blind.

## Supplementary information

supplemental material

## Data Availability

All experimental data and details of this study are available are available from the corresponding author upon request.
