# Supplementary material for: Syntabulin regulates neuronal excitation/inhibition balance and epileptic seizures by transporting syntaxin 1B
Source: Cell Death Discov. 2023 Jun 22;9:187. doi: 10.1038/s41420-023-01461-7 (PMC10287671; doi:10.1038/s41420-023-01461-7)

**Table 1.Clinical characteristics of epileptic patirnts**

| Case | Gender  (M/F) | Age  (years) | Courese | AEDs before surgery | Resection tissue |
| --- | --- | --- | --- | --- | --- |
| EP 1 | M | 25 | 11 | PB,VPA,LTG | RTN |
| EP 2 | F | 21 | 8 | TMP,VPA,CBZ | LTN |
| EP 3 | M | 39 | 10 | TPM,VPA,CBZ | LTN |
| EP 4 | M | 18 | 15 | PHT,PB,VPA,CBZ | RTN |
| EP 5 | F | 26 | 7 | TPM,VPA,CBZ | RTN |
| EP 6 | F | 20 | 6 | TPM,VPA,CBZ | LTN |

EP=epileptic patients; F=feamle; M=male; AEDs=antiepileptic drugs; PB=phenobarbital; VPA=valproate; LTG=lamotrigine; TPM=topiramate; CBZ=carbamazepine; PHT=phenytoin; RTN=right temporal neocortex; LTN=left temporal neocortex.

**Table 2. Clinical characteristics of control individuals with brain trauma**

| Case | Gender  (M/F) | Age  (years) | Mechanism of injury | Resection tissue | Neuropathological diagnosis |
| --- | --- | --- | --- | --- | --- |
| TBI 1 | F | 41 | Accident | RTN | N |
| TBI 2 | M | 15 | Accident | LTN | N |
| TBI 3 | M | 21 | Fall | LTN | N |
| TBI 4 | F | 25 | Accident | RTN | N |
| TBI 5 | M | 18 | Accident | LTN | N |
| TBI 6 | F | 17 | Accident | LTN | N |

TBI= traumatic brain injury; M=male; F=female; LTN=left temporal neocortex; RTN=right temporal neocortex; N=relative normal.

**Fig 1**


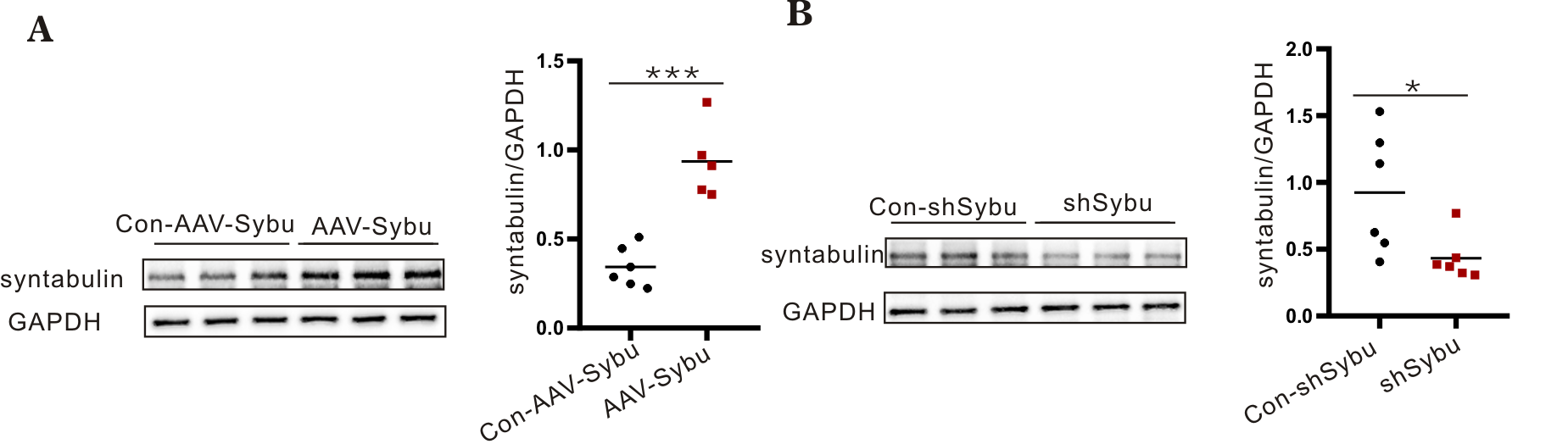


**Fig 2**


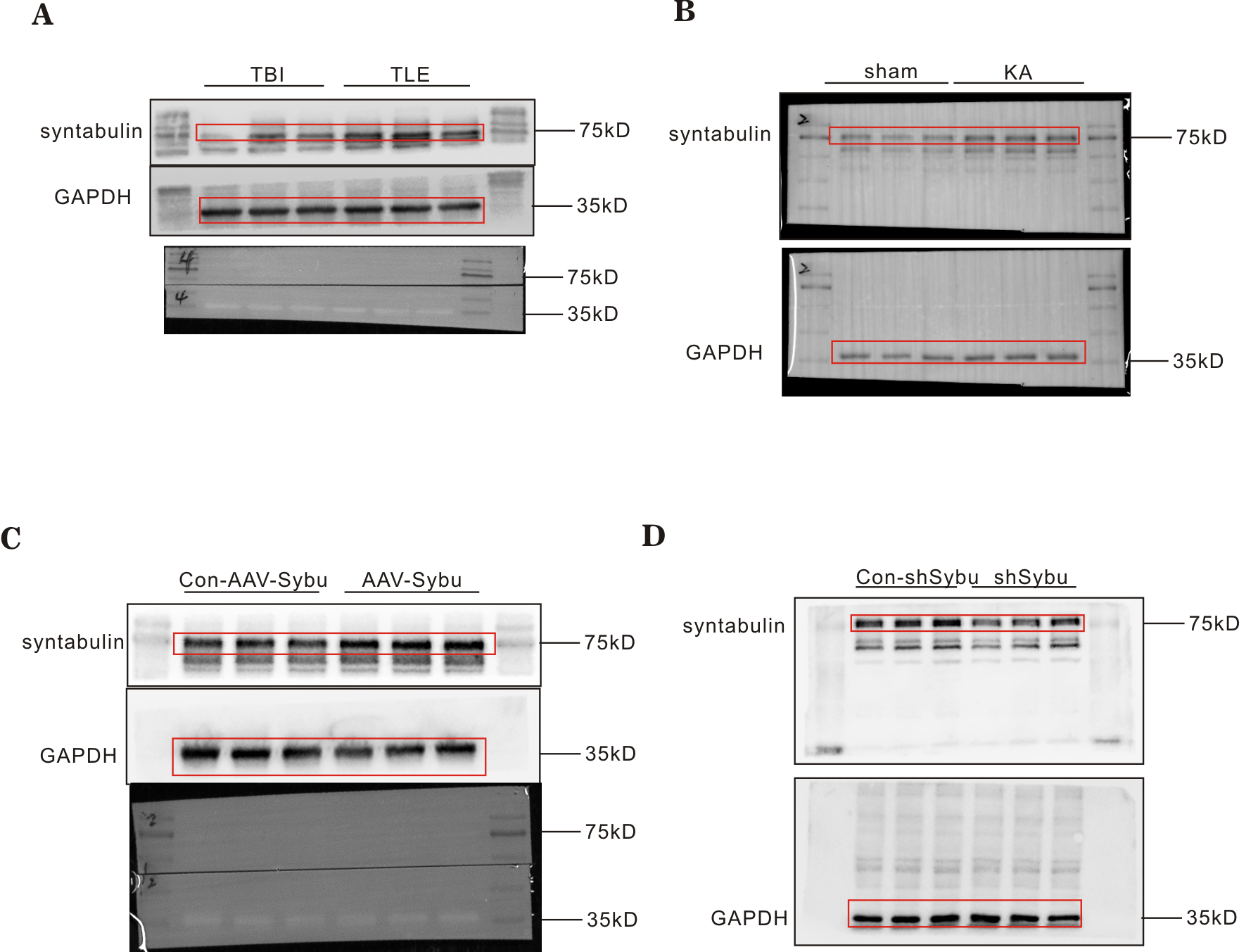


**Fig 3**


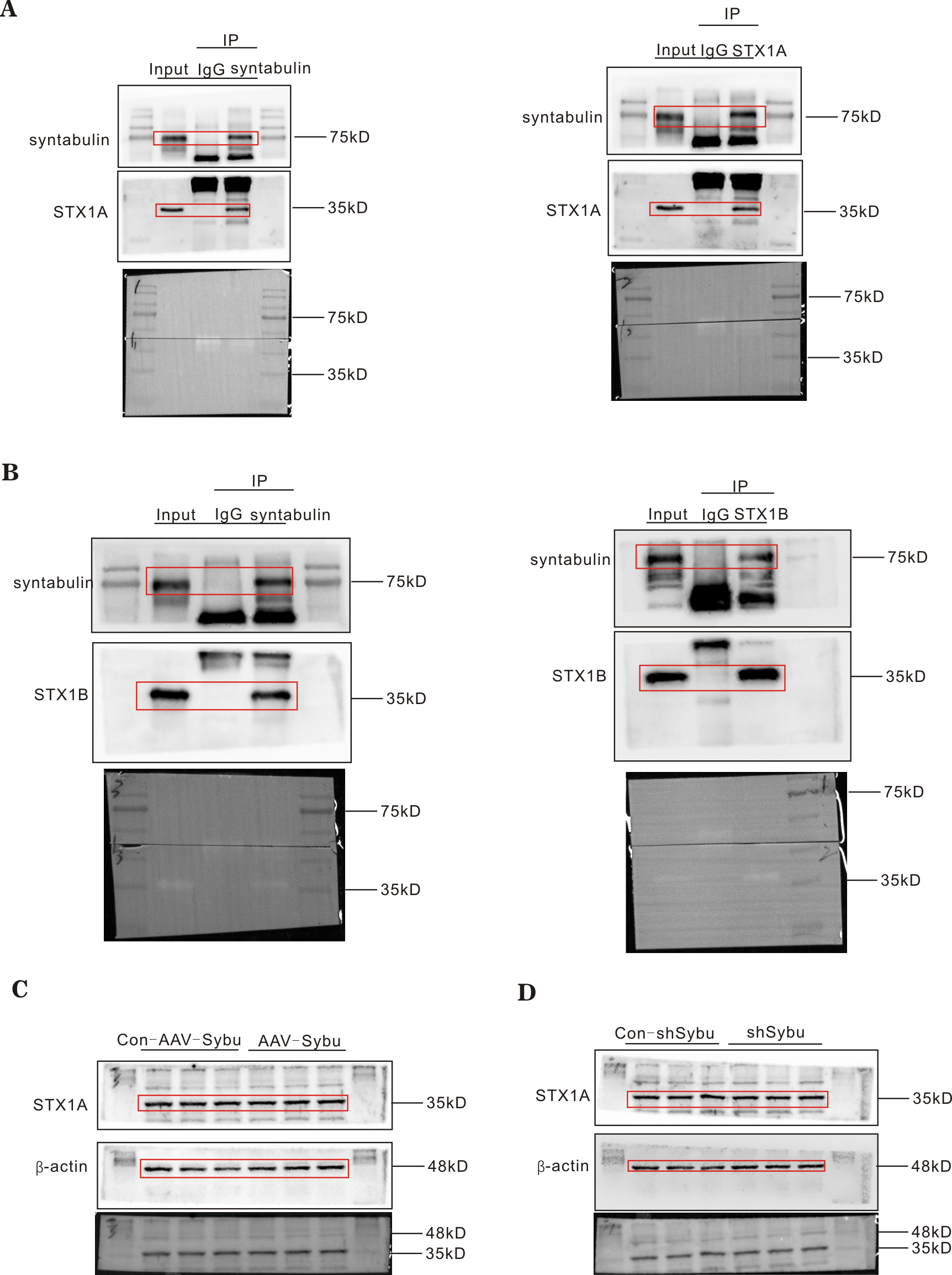


**Fig 4**


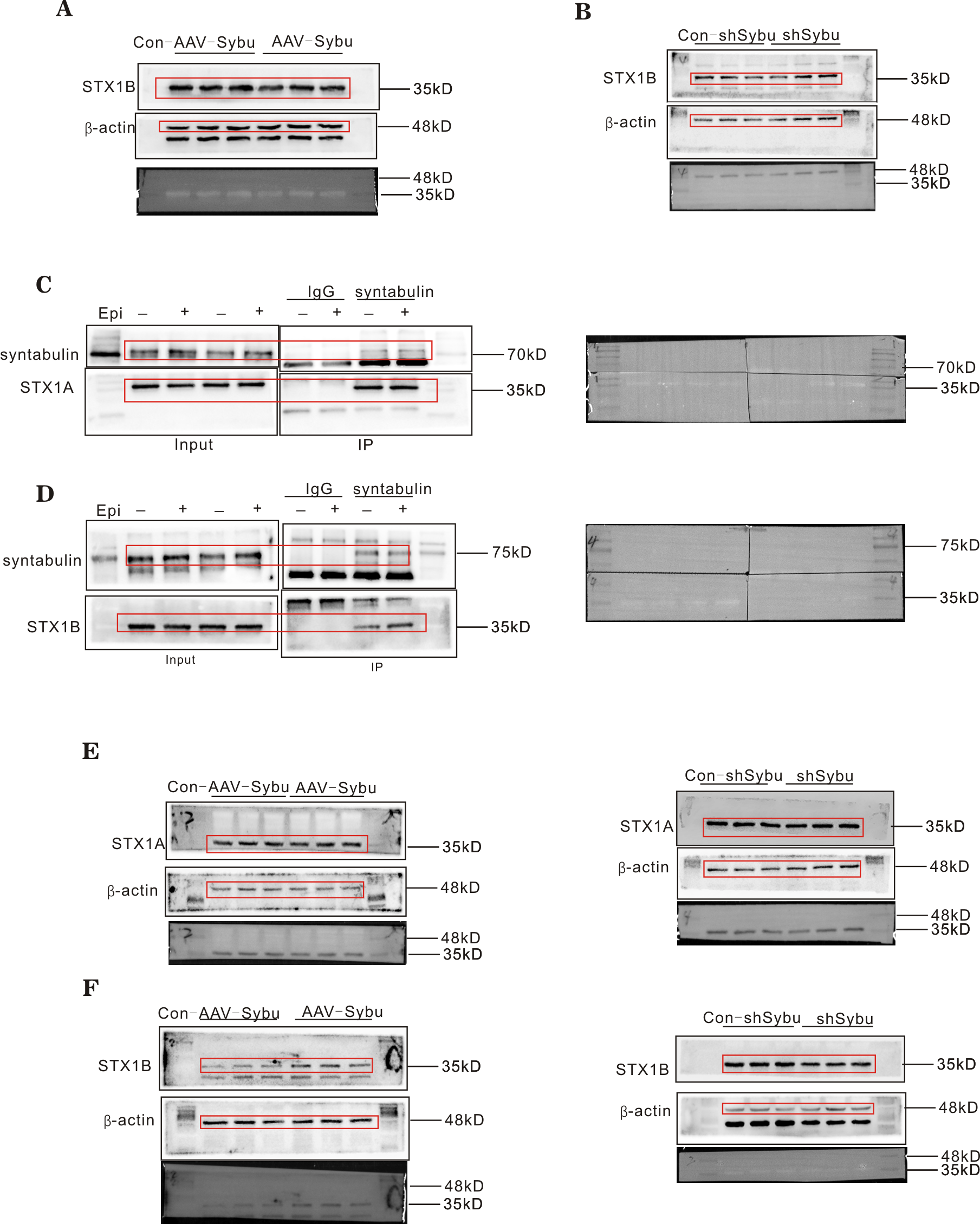


**Fig 5**


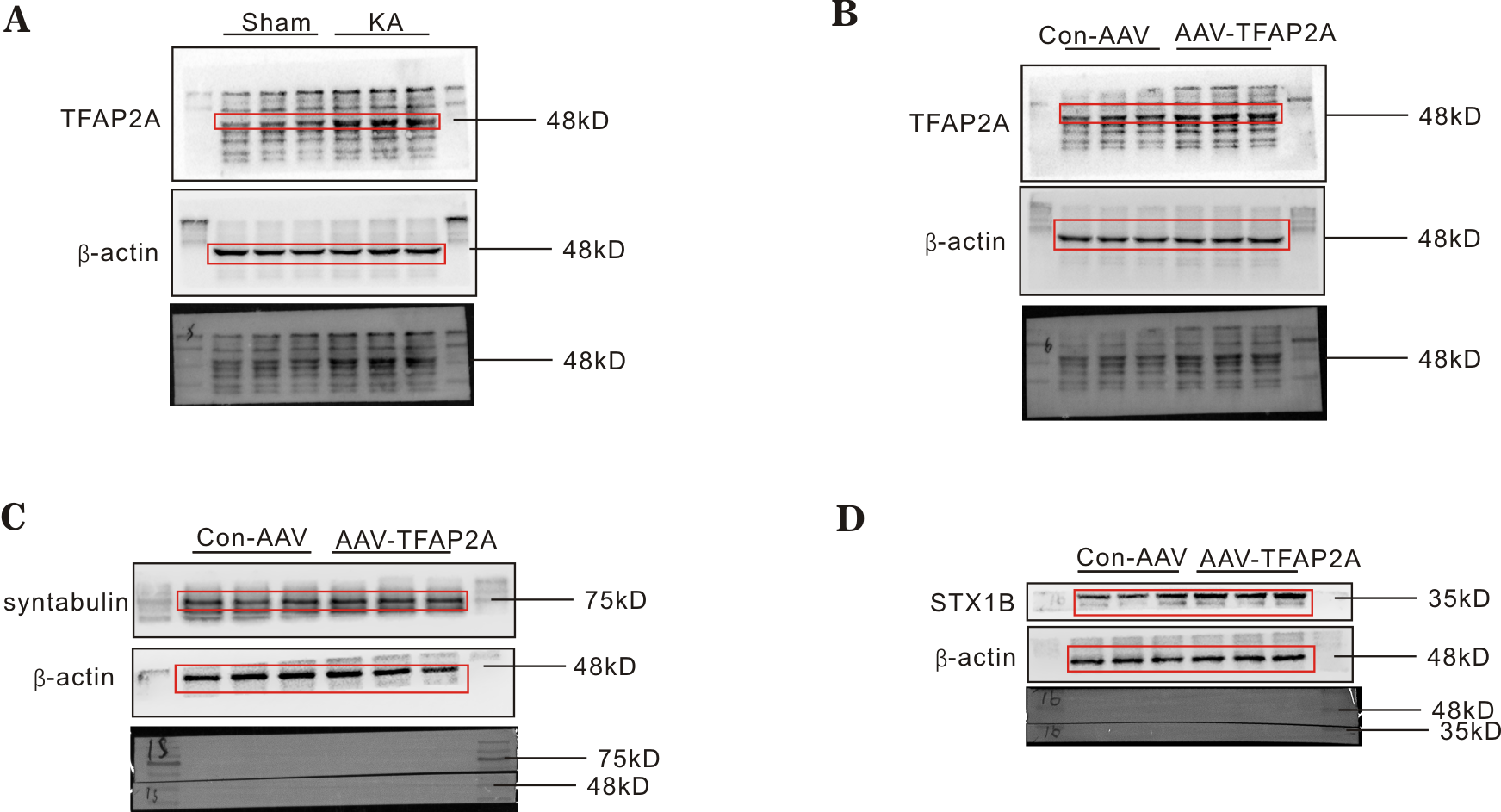

Supplement: Supplementary file 1 — supplemental material [file 41420_2023_1461_MOESM1_ESM.doc]
